# Observations on Mental Derangement

**Published:** 1832-04-01

**Authors:** 


**Observations on Mental Derangement; being an Application of the Principles of Phrenology to the Elucidation of the Causes, Symptoms, Nature, and Treatment of Insanity. By *Andrew Combe*, M.D. 12mo. pp. xxxvi, 392. Edinburgh and London, 1831.**

Whosoever desireth to retrace the movements of an ingenuous mind through the stages of a candid and philosophical investigation of doctrine, may derive instruction from a fair perusal of Dr. Combe's introductory statements: we advise all students of physiology to examine these statements, and the *note* appended to them, with a spirit disengaged from every prejudice, and unembarrassed by the hebetating obliquities of preconception.

"" Dr. Combe's "*Observations*" are distributed into *ten* chapters: in our analysis of his book, we shall adopt the same arrangement.

Chapter I.—*General Remarks on the Functions of the Brain and Nervous Sys-*

*tem*. Dr. Combe receives, as self-evident, the proposition in physiology, that every distinct organ in the animal economy performs an appropriate function; and, as a necessary consequence, that every distinct function is executed by an appropriate organ; hence, wherever a plurality of functions has an obvious connexion with a part, that part may be regarded as having a compound structure. On this principle, therefore, whenever we discover in any animal, either a new organ, or an addition to an organ previously ascertained in other animals, we may conclude that a new function, or some modification of, or addition to, a function already known, remains to be distinguished; and that, on the other hand, where we perceive a new function, or a modified state of an old function, there we may infer the existence of a new organ, or of an additional portion of organ, by which the new function, or modification of function, is exercised. Many years ago, Dr. Gall began to apply this principle, practically, in his researches concerning the cerebral organs; and, by this means, evinced how it can be employed in elucidating the anatomy and physiology of the brain. Guided by this original principle, indeed, he completed the demonstration, by arguments, analogies, and facts innumerable—that the brain is an aggregate of parts, each of which resembles the rest in substance and structure, but is nevertheless essentially distinct, and endowed with a naturally different function.

Many circumstances, in health and disease, had concurred to induce a general belief, even in very remote times, that the cerebral mass comprises an aggregated system of organs, every one of which fulfils its own peculiar office: Gall, however, was the first to convert this belief into a certain position; indeed, the different cerebral terminations of nerves, seem sufficient to imply a diversity of function in the different parts of the brain with which its proper nerves are associated. Similar reasons, in like manner, render it probable that the apparently homogeneous nervous bundles are complicate, with each elementary part having a peculiar determinate use. This notion had long been entertained by reflecting men; but it was first promulgated as a principle, about twenty years ago, in being submitted for judgment to the French Institute and the scientific world, by two physiologists, who have merited a just and honorable reputation by their discoveries in the anatomy and physiology of the nervous system: their original perception of this principle suggested those philosophical inquiries, from which has resulted—our knowing the natural divisions of the nervous tissues and the plurality of their functions. Thus, they said—"it can happen only, when anatomy and physiology are founded on each other, that our knowledge of the nervous system will attain the highest degree of perfection."* Again—"evidently the nervous system is not simple and uniform; it should be divided according to its *chief* functions, and each of its principal divisions ought to be subdivided according to its *particular* functions."† Again, "the nervous systems differ from each other in their origin, their structure, their colour, and their solidity."‡ Again, "nerves differ from one another in the variety of their configuration; thus the nerves of the senses have no resemblance, one with the other, in their colour, their consistence, their form and their texture: often, the different filaments of the same nerve are quite visibly dissimilar: not only do the different nervous systems, but also the different fibrils of a nerve even, arise from distinct portions of the grey substance situated in different places; all these peculiarities are the same in the same nerves; hence, as a cause, there must be a primitive difference in their internal structure, and, by necessary consequence, an essential diversity in their functions."§ Again, "the nervous system must be divided and subdivided: each part of these divisions and subdivisions has its

---

* Anatomie et Physiologie du Système Nerveux en général, et du Cerveau en particulier, par les Drs. Gall et Spurzheim, 1810. Tome I. p. xxii.

† Ibid. p. 11. ‡ Ibid. p. 75. § Ibid. p. 128.

*peculiar origin.*"* Again, once more, "there is a difference in the nerves of motion, and those of feeling, because the same nervous fibres do not go to the muscles and to the skin, and each of these parts performs peculiar functions; the nerves necessary to motion, cannot propagate the impressions of the sentient nerves, nor these the impressions of the nerves of motion: from observations, anatomical and physiological and pathological, we infer that the nerves of motion and feeling are different."† Adopting or discovering this principle, Sir Charles Bell and Dr. Magendie applied it successfully in their experimental researches to ascertain the primary differences and functions of nerves; and, by many conclusive evidences, made a perfect demonstration of the fact—that the nerves of sensation and those of motion, are as distinct in their origins and their distribution, as in their offices.

Dr. Combe, at p. 7, illustrates the doctrine clearly,—that, since the brain is the centre to which all external impressions must be conveyed before *sensation* can take place, and from which all *volition* must proceed; therefore, every nerve which serves to conduct these impressions *to*, or to transmit the commands of the will *from*, the mind to a distant organ, necessarily requires to be in free communication with the brain, either by direct origin from some one of its constituent parts, or by the medium of the spinal cord, with which some parts of the brain are intimately connected, both in structure and function. Hence, therefore, to effect *sensation* or the *perception* of impressions made on the sentient extremities of the nerves, a consentanëity of action, and a freedom of communication between them and the brain, are indispensably requisite. By the right application of this principle, we shall *often* be able to retrace, to distant organs, the essential causes of cerebral disturbance.

These remarks, the Doctor continues, apply to all the nerves of animal life, internal as well as external: it is always the brain, not the nerve itself, that feels the affection; of this last, the nerve is only the conductor. On this account, then, the action of the brain is modified by every change in the condition of the nervous fibres ramified on every part of the body: hence, consequently, a reciprocal communication and influence are essential to the due exercise both of the brain and nerves. He adds illustratively, p. 9.—

"The brain, being truly the seat of sensation, it happens, that if, by any internal cause, such as disease, that part of it which is in correspondence with a nerve, be excited in the particular way in which it is acted upon by the natural stimulus from without, the same sensation will arise in the mind as if the external were actually present, and making its usual impressions on the nervous filaments, and a belief of its real existence will accordingly take place, unless the mind be informed by other faculties of the source of the error. Such, accordingly, is the origin of the many odd feelings,—of the cravings of hunger without real want of food,—of the creepings under the skin,—of the voices constantly whispering into the ear—of the deceptions of sight,—and of the all-pervading smells and tastes so commonly complained of in madness, and sometimes also by persons in sound mind; the only difference being, that the former believe in the existence of an external cause to excite those sensations; and that the latter perceive their morbid origin, and refuse to obey their impulses."—These observations comprize much practical instruction: so, likewise, do those that follow. "In health," says Dr. C., p. 10, "the stimuli to which the nerves are adapted, and of which they transmit impressions to the brain, are so perfectly in harmony with their constitution and structure, that their action is carried on almost unconsciously. But, if disease attacks the organ or part on which they are ramified, the state of things instantly changes: the nervous filaments,

* The Physiognomical System. By J. G. Spurzheim, 1815, p. 13, 14.
† Ibid. p. 23, 24.

stimulated perhaps to excess by great irritation in the part diseased, then transmit to the brain the same excess of stimulus which they receive, and rouse the cerebral part with which they are more immediately connected into inordinate action : and, if the irritation be of long continuance and considerable severity, and the organ be one strongly connected with the brain by a multiplicity of nerves, or the importance of its function, the inordinate cerebral action thus induced may so far affect the structure and functions of the brain itself, as to excite in it a morbid action, which will go on independently of the cause which produced it ; and this is in reality one of the sources of cerebral and mental disease."

Dr. Combe now arrives at the following important conclusions. 1st. That, as every single part is capable of performing one function only ; and, as every system of nerves arises from corresponding nervous masses performing functions analogous to its own ; it follows, that the higher we rise in the scale of living beings, and the more numerous the functions with which each is endowed, the more perfect and the more complicated will their nervous organization become. 2d. That, when we consider the number and perfection of the senses, and the manifold propensities which man possesses in common with many animals, having distributed among them what are united in him, and the various moral and intellectual faculties which pertain to him exclusively—the natural and irresistible inference is, that he must have a brain and nervous system complicated and perfect in structure, in exact proportion to the higher perfection and more numerous endowments which the Creator has bestowed upon him. 3d. That, on resorting to observation, we discover in confirmation of these inductions—that, in precise proportion as we ascend in the scale, and the animal acquires a sense, an instinct, a power, so do its nerves multiply, and "its brain improves in structure, and augment in volume, each addition being marked by some addition or amplification of the powers of the animal, until in man we behold it possessing some parts of which animals are destitute, and wanting none which they possess," hence, *we are enabled to associate every faculty which gives superiority, with some addition to the nervous mass, even from the smallest indications of sensation and will, up to the highest degree of sensibility, judgment, and expression.** Being, thus, at once the seat of intellect and of instinct, the centre of sensation, and the chief source of nervous energy, and therefore a complex organ, the brain is admitted, by almost all modern physiologists, to receive successive additions in different animals as they ascend in the scale of creation, and become endowed with additional instincts. Animals of the lowest class, which manifest no traces of sensation, have neither brain nor nerves ; but wherever a *will*, a *rational will*, depending upon a choice between different impulses or motives, exists, there a brain exists also ; and this becomes complicated and perfect in proportion to the number of instincts or faculties with which the animal is endowed. It appears, indeed, the Doctor adds, that some of the lower animals have particular parts of the brain more fully developed than these parts are in man ; at the same time, however, they have particular senses more acute, and particular instincts more powerful, in correspondence with these particular parts :—but, then, no animal has a brain consisting of so many parts, or so fully and perfectly developed, as is that of man ; and no animal possesses so many powers of feeling and intellect as those which constitute the human mind. Finally, here, the simple fact—that mind is incapable of manifesting itself except through the instrumentality of brain—is, in one sense, logically sufficient to settle this question ; for, if the human brain did not comprize parts which are inexistent in the brains of ani-

* These remarks, in Italic letters, are taken from the Edinburgh Review, No. 94, p. 442, 3 : having transcribed them, Dr. Combe exclaims, in amazement, "Who could have expected to find such doctrine in such a place ?"

mals, it would be able to manifest those qualities only that man and animals have in common :—just as, without eyes, man could not enjoy vision ; or without ears, execute hearing, however perfect the internal or incorporeal constitution of his mind might be.

According to our author, the fundamental principles of the new physiology of the brain are—1st. That the mind is endowed with a plurality of innate faculties ; 2d. That each of these faculties manifests its own affections, through the instrumentality of an appropriate organ, of which organs the brain is a congeries, aggregate or system ; 3d. That the *power* of manifesting its affections, possessed by each faculty, bears a constant and uniform relation, CÆTERIS PARIBUS, to the *size* of the organ or part of the brain with which it is more immediately connected ; 4th. That it is possible to ascertain the relative size of these different organs during life, by observing the different forms of the skull to which the brain gives its shape. By most physiologists, the first and fourth of these propositions are admitted. Dr. C. in his first chapter, supports the second by the strongest *probable* evidence ; and we can obtain the *demonstrative* only by observation in Nature. He regards the third as having a very important practical relation to the pathology and treatment of insanity. We propose to accompany him in his discussion of this proposition ; and to examine with him its direct and analogical evidence.

CHAP. 2.—*Influence of Organic Size on Energy of Function, particularly as applied to the Organs of the External Senses and Brain.* We are sorry that we must pass over this chapter untouched, though it contains much interesting matter.

CHAP. 3.—*Mental Derangement is always Symptomatic of Cerebral Disease.* Many very wise, and some very witty, persons occasionally seek a diversion to their idleness, in ridiculing the uncertainty of opinion and the inconsistency of doctrine with which medicine is too often petulantly charged. Nevertheless, when their own lives come into danger, these persons solicit assistance with an alacrity quite indicative of their serious belief—that medical science has a foundation in the nature of things and that its professors possess an extent of certain knowledge, by the right application of which the lives of mankind may be prolonged, and their miseries greatly mitigated. From this fact, the conclusion is, that the imputed inconsistency does not arise from the absence of invariable and permanent principles, according to which the various functions of the human body are regularly carried on, and according to which their disorders ought to be treated ; but the inconsistency arises entirely from our imperfect acquaintance with these principles, and with the numerous modifications which they undergo, from the influences to which man is exposed : consequently, in proportion as our knowledge shall become more extensive and accurate, so will the contradictions and inconsistencies which have brought obloquy upon medicine gradually disappear. It is encouraging, then, to *know* that, in pursuing medical inquiries, our difficulties are ascribable chiefly to the latter cause—and *to feel* assured that the mental and bodily constitutions of man did not come from the CREATOR's hands undefined or imperfect ; and that all the animal functions are regulated by fixed and determinate laws, and have fixed and determinate relations to every class of external objects. Now if this view be correct, and if the causes of disease, and the agents employed for their prevention and cure, thus have definite properties, and act upon a system regulated by definite laws, *then* medical science must necessarily advance concomitantly with the progress made in the discovery of these laws and of their relations to each other :—thus every new error into which we may fall, instead of deterring us from pursuing our investigations, becomes a new beacon to guide us past some of the dangers to which we were formerly exposed ; whereas, if medicine were an art without principles permanent as Nature herself, its advancement would be as hopeless a task as ever attracted and deluded the ingenuity of man. A conviction that medicine rests upon fixed principles, Dr. C. observes, is nowhere more valuable, than as applied to the diseased manifestations of mind: for instance, it is an established principle in pathology, that every derangement of *function* is always accompanied by a disorder, either in the structure or in the mode of action, of the *organ* which performs it, and without the removal or cure of *which*, the *function* cannot be restored to its healthy state. Acting on the faith of this law of the animal economy, we almost instinctively begin our examination of a patient, with an endeavour to find out what functions are chiefly vitiated; through them, we go back to the organs which execute them; and *there*, by local and other symptoms, we seek the kind of disease which has caused the aberration of function:—by following this procedure, we often succeed in ascertaining the seat, nature, and method of cure of the disease requiring our attention. When, however, we look to the notions and modes of proceeding which have long prevailed in regard to insanity, we often see a melancholy reverse of the picture. From ignorance of the *fact*, or from want of confidence in the *fact*, that the *principles* of medicine are *immutable* and *permanent* in their operation, many persons are contented with viewing the disjointed phenomena of mental derangement as the inscrutable consequences of an affection of the immaterial principle of mind, or as a particular dispensation of Providence, which they could not be expected to understand or to remedy: accordingly, while this view continues to influence practice, all sorts of incongruous measures will be adopted against the miserable sufferers, and the short fit of their frenzy be too often converted into irremediable mania or hopeless fatuity. Were, however, the law of the constant connexion between the state of an organ and the mode of its function more intimately known, *and the* universality *of its application confided in*, it would lead the observer to investigate the condition of the organs, whose function it is to manifest the mental faculties—to look *there* for the seat of the morbid action, and thus to determine its nature and treatment on rational, inductive, and consistent principles:—that the brain manifests the mind's faculties, is a proposition clearly demonstrated; hence, the true pathology of insanity is to be sought in the history of the various diseases to which the cerebral structure is liable.

"Knowing nothing of the mind's nature," says Dr. Combe, p. 65, "and, in this world, having no means of knowing any thing of its *nature*, as it exists independent of, and separate from, the organization with which it is connected during life, we can only study the capacities and modes of action which it exhibits in its combined or compound state; and, to attempt any thing beyond this, would be not only unnecessary, but utterly useless labour. We cannot reach the *principle* of mind, to modify its qualities or manner of being; we can reach it only as acting through the medium of, and influenced by, its material instruments; and, consequently, all attempts to improve its powers, and to extend its limits, must be conducted with a constant reference to the organic conditions under which it acts, otherwise they will to a certainty fail of success. During life, indeed, the closest relation obtains between the mode of action of the various mental powers and the condition of their respective organs—every change in the state of the one being always accompanied by a corresponding change in the state of the other. All the faculties of thought and of feeling are feeble and inefficient in infancy—not from any defect in the immaterial principle of mind, but simply from the imperfectly-developed condition of the organization which, *in this life*, is required for their adequate manifestation. Some animals see distinctly immediately after birth, but hear very imperfectly; others hear, but do not see; and others, again, are almost insensible alike to sounds and to vision. Every body knows the explanation of these facts; thus, in one animal, one *organ* of sense is early developed; and, in another, a different *organ* is first matured. In infancy, in like manner, some *internal* faculties, the *organs* of which are early developed, precede in maturity others, whose *organs* are not fully developed till much later in life. In youth, the *observing* powers preponderate in energy and activity, and the corresponding cerebral *organs* bear a visible predominance over those of the *reflecting* faculties which come later to maturity—thus demonstrating, at every step, the intimate connexion between the mode of action of every faculty, and the condition of its *own* material organ. If we look at the mind as a *whole,* we shall find it following the same rule of progression : in infancy, the mental powers are feeble and vacillating in their exercise ; vigorous and enduring in manhood ; and again deficient in energy and vivacity in old age—in exact correspondence to the progressive changes in the organization of the brain, from that of very imperfect structure in infancy, to that of progressive maturity and decay, as occurring successively in youth, in manhood, and in old age. Moreover the effects of fatigue, and the necessity of sleep for recruiting the mind as well as the body; the changes in thinking and in temper caused by the corporeal states of repletion and of hunger ; the effects of opium and alcohol; the changes, the almost total abolition at one time, and astonishing excitement, force, and irregularity of action at another, of the different powers of the mind, in consequence of bodily disease or of accidents, shew incontestably the never-ceasing dependence, *in this world,* of mind upon brain for its manifestations. Not only, however, do the mental powers follow the regular and comparatively durable changes thus brought about in the condition of their respective organs ; but they are also affected, in an equally evident manner, by every change, however slight, and of however short duration, to which the organization is subject, either from external or internal causes. The touch of a hair upon the skin, the falling of a single ray of light upon the eye, or of a single atmospherical pulse upon the ear, are sufficient to cause corresponding changes in the state of the mind. Sudden compression of the brain is well known to deprive the patient of all mental power ; and it has even happened, again and again, that where an opening existed in consequence of a fracture of the skull, by pressing the brain with the finger, consciousness was destroyed, to be restored on removal of the preſsure : and repetition of the experiment was attended with precisely the same results."

Any organic part may be brought into a morbid state, by causes acting directly upon its function, or by causes immediately affecting its substance ; thus, inflammation of the eye may be excited either by stimulating its function with too much light, or by sand, or lime, or cold air affecting its surface. Now the brain offers no exception to this law: the fact, indeed, explains how derangement of the mental faculties came to be considered apart from its corporeal cause. One person, from a reverse of fortune, great affliction, disappointed love, or intense study, becomes insane or delirious, with symptoms of a cerebral affection —this presents an instance of excitement of function inducing disease of the organ. Another person, from an injury, from a *coup-de-soleil,* or from intoxication, falls into the same state ; this is an example of the same result being consequent on the direct application of external influence to the organ itself. Now the true relation between the two states was not sooner perceived—*because* it was always forgotten that the function of the brain is to manifest the mind ; and that, in so far as the manifestations of the mental powers are concerned, the agency of the brain is as indispensible as if it were the mind itself. These facts bear upon the subject of imperfect or disordered manifestations of the mind : thus, hitherto, a singular and unfortunate distinction has been made between derangements of the *external* and those of the *internal* faculties of the mind ; the external organs of the senses having long been known, we justly ascribe every disturbance of their functions to an affection of their material organs, and our efforts are directed to the discovery of the nature of the particular affection then existing ; and by this discovery our treatment is regulated : but, when an internal faculty of feeling or of thinking becomes deranged, *instead of following the same rational*

*course and ascribing its aberration to an affection of its cerebral organ*—we generally content ourselves with the simple but vague affirmation—*that the mind is deranged*, and have not cared to discover the particular organic cause of the disturbance of function. Between the external and the internal faculties, there is really no greater difference than between one external sense and another: all are equally powers of the mind; they differ only in having different functions to perform, and in each having an organic instrument fitted for the exercise of its specific function: thus, to enable it to see, the mind requires an optic nerve with an external eye, *because* light is an external existence with which it must be connected; and, in like manner, the mind requires an internal cerebral organ to *feel* the sentiment of justice, *because* justice is not an external quality, but a mental or internal relation: again, the mind requires an external organ to enable it to hear, *because* the vibrations of the air are external existences with which it must be connected; and, in like manner, the mind requires an internal organ to *feel* the sentiment of pity, *because* pity is not a quality of matter, but simply a mental state or relation. From all this, it is manifest—that the organs of the five *senses* are merely parts added to the other cerebral organs in order to connect the *perceptive* faculties with the external world: the powers of *seeing, hearing, tasting, touching* and *smelling*, are neither more external to, nor less intimate parts of the mind, than any other of its powers; and the well-being of the brain is alike necessary to the right exercise of them all. Evidently then, if the manifestations of the mental faculties, in a state of health, depend on the healthy condition of their organs, external and internal, and if a change in the state of the mind attends even the slightest alteration in that of the brain —*it follows*, that a morbid condition of the organ of mind must be attended with disordered manifestations or mental derangement,—and that, without the previous removal of this organic cause, the mental health can never be re-established.

Such doctrines as the foregoing, although enforced by observation and confirmed by experience, are denounced by the unthinking and the prejudiced as dangerous to religion: by such persons it is argued—that to refer insanity to an organic cause, is to confound mind and matter together; to teach that the brain *is* mind; and, consequently, to destroy the strongest proof of the soul's immortality. Happily for humanity, however, truth and reason are as imperishable as mind; and, under their influences, it is now widely acknowledged, —that it is the old and false doctrine of the *mind* being subject to *disease*, which is justly chargeable with the apprehended danger,—and that, if the soul's immortality can be proved in any way by *reason alone*, this proof must rest exclusively on the grounds here advocated. The relation thus shown to exist between the state of the mind and its *material* organs readily explains WHY—*the immaterial principle remaining essentially unchanged*—the mind develops its powers as we advance from infancy to manhood and declines as we descend from manhood to old-age,—WHY it falls asleep in the night, or loses consciousness from a blow on the head,—WHY its manifestations are disturbed by intoxication, or deranged by disease,—and WHY it is characterized in *one* by the weakness of idiocy, and in *another* by the strength of genius. Moreover, the facts—that the mind never manifests itself in this world except through the *instrumentality* of corporeal organs, and that the *condition* of these organs influences the *quality* of the manifestations—afford an easy explanation of the origin of mental derangement and of the possibility of its occurrence without endangering the mind's *essential principle*. Through the *instrumentality* of the eye and the optic nerve and a portion of the brain, the mind *sees*, just as it *thinks* or *feels* through the *instrumentality* of other portions of the brain: and, as changes in the *condition* of the organs of sight deteriorate or destroy the *power* of vision *without any affection of the* principle *of mind*; so, in like manner, do changes in the *condition* of the brain derange or destroy the power of *feeling* or of *thinking*, and still the mind itself remains essentially unimpaired. On the other hand, if we ascribe the varying mental states to variation in the *immaterial* principle, unconnected with any corresponding organic cause, then must we hold also—that the defective mind of the idiot from birth, has been purposely created thus mutilated and limited in powers—that it is the mind itself, and not the body, which is disordered by wine, set to sleep by opium, and apparently annihilated by a blow, though the wine and the opium and the blow all act perceptibly on the *body*,—that it is the mind itself, and not its corporeal organ, which is weak in infancy, strong and active in maturity, and again feeble in old age,—that it is the mind itself, and not its corporeal instrument, which is subject to delirium in fever and to the manifold other forms of disease which impair or derange or suppress the manifestations of the mind's constituent faculties. Admit we all this, and at what point are we to stop? If the *soul*, the immaterial principle, be thus subject to disease and to *apparent* annihilation and to changes interminable, it is impossible to draw any evidence of its immortality from *reason alone*: but, on the view that it is the mind's *organ* which suffers disease and disturbs the mind's *manifestations*, the doctrine of the soul's immortality remains open to every *rational* proof that can be urged in its favour.

Mental derangement, then, originates in a disordered state of the brain's functions: this state arises from some morbid action in that organ, and may or may not involve, at the same time, the functions and organs of the external senses: frequently, however, it exists without any such complication: this morbid action must be remedied before the mind's alienated manifestations can be removed. Dr. Combe, on p. 74—5, renews, concentrates, and enforces the evidences by which his fundamental proposition is demonstrated: thus, "by constantly drawing attention to the connexion subsisting between the *power* of manifesting every mental faculty, and the *condition* of its particular cerebral organ, an enlightened physiology places derangement of the mind's internal faculties in the same relation to the organic affection producing it, as that wherein it places the derangements of the five external senses. *Sight* is never impaired nor *hearing* destroyed, unless the organs which execute these functions, are diseased; in like manner, *thought* and *feeling* are never deranged, unless the cerebral organs by which they are manifested, have undergone some morbid change; and as sight is injured by a great variety of morbid alterations in the eye or its nerves, so are the internal faculties of the mind deranged by a great variety of diseases affecting the brain. Even if we had not *direct proof* of the dependence of mental derangement on various cerebral affections of a different nature, the force of *analogy* is still so strong as of itself to establish the fact, and to satisfy the most sceptical enquirer—*that insanity is not a single and unvarying disease*. Every affection to which an organ is liable may derange its function, and therefore disturbance of the functions of the brain may attend a variety of different cerebral states, each characterized by its own symptoms and requiring its own mode of treatment. The eye, for example, is the external organ of vision, and any affection of the eye—*whatever its nature*—may derange its function and impair sight. The eye may be inflamed, or it may be distended with water, or opacity may cover its convex surface, or the optic nerve may be paralysed; and, as a consequence of *all* these states, impaired vision or blindness follows: impaired or destroyed vision is therefore not a specific disease, but a proof or symptom of the existence of some affection having its seat in the organs of sight, the real nature of which must be determined by other means. The ear is the organ of hearing; and all affections, of whatever nature, having it for their seat, may injure its function: the ear may be inflamed, or the tympanum may be ruptured, or the acoustic nerve may be paralytic; and, as a consequence, hearing may be destroyed: this also shows that impaired hearing and deafness are not diseases, but merely symptoms attending maladies which have their seat in the ear. The lungs are the organs of respiration; and all causes, of whatever nature, affecting them, may derange their function and impede breathing: the lungs may be inflamed, or may be the seat of an extravasation of blood, or they may be compressed by water or air in the chest; and, as a consequence, in all these cases, respiration may be impeded; so that dyspnœa or difficult breathing is not a disease by itself, but merely a symptom attending diseases which have their seat in the lungs. In like manner, the brain is the organ of the mental faculties; and any affection of whatever nature, having the brain for its seat, may disturb the cerebral function, or the manifestations of the mind: the brain may be inflamed, or it may be excited by wine, or compressed by water or by a fracture of the skull; and, as a consequence in all these cases, the mind's manifestations would be disturbed. Derangement of these manifestations, therefore, is not a disease; it is a symptom attending many different affections, which agree only in the single point of having the brain for their seat."

After shewing the practical utility of founding treatment on these views, the Doctor proceeds to say, p. 77—"But had the fundamental principle, that the brain is the *organ* of mind, and, consequently, the fact, that insanity always depends on a corporeal and cerebral cause, been recognized and kept in view, it would have at once been perceived that, as every departure from health in an organ must necessarily disturb its function in a greater or less degree, and, as the function of the brain is to manifest the mind, mental derangement could not be a specific disease, but must be one of the effects of whatever morbid causes disturb the action of that organ, and could, therefore, no more be considered as an individual disease than impeded respiration, impaired vision, or vitiated secretion of bile. Had the attention of the observer been closely directed to the study of the relations subsisting between the mental faculties and their cerebral organs, so many centuries could not have elapsed, and so little been added to our knowledge of a subject, in which mankind at large is so nearly concerned. Had insanity been recognized as a symptom of cerebral disease, the insane would never have been rejected and excluded from our sympathies as the detested of Heaven, nor would they ever have been tortured by the lash or the chain, or exposed to public derision. Had a glimmering of its true nature reached the public mind, we would as soon have thought of loading the gouty or the paralytic with reproaches and obloquy, and of curing them by the application of the bastinado, as of treating the maniac with the neglect, and often positive cruelty, which he once met with. The moment we know that madness is an effect of disease in the material organs, with which the Creator has connected the *principle* of mind, and that to this *infliction alone* are to be ascribed the waywardness, violence and impetuosity, which often characterize that state, our feelings towards the unhappy patient, and our attempts at cure, will be very different indeed from what they would be, were we still ignorant of its true nature."

Dr. Combe divides the affections of the brain which disturb the manifestations of the mind into two primary classes—1. Those diseases which are ACUTE in their character, rapid in their progress, and dangerous to life; such, for example, are fevers, phrenitis, hydrocephalus acutus and apoplexy; 2. Those which are CHRONIC in their nature, slow in their progress, and compatible with a prolonged existence; such, for instance, are the various maladies which give rise to insanity. His remarks on this division are most instructive; thus, he observes—In the former, from their being attended with local symptoms of great intensity, the derangement of the feelings and intellectual powers is universally and readily ascribed to morbid changes in the brain; but, in the latter, where the symptoms are not so severe, and where the disturbance of the mental operations is equally manifest, though sometimes differing in character, the same connexion of the phenomena with their cause in the brain, is frequently not only unperceived, but resolutely denied. As it is of the utmost importance, in practice, to be aware of the relation subsisting between the two *classes* of cerebral affections—*that the obscurities of the one may be relieved by the lights afforded by*

*the other, and that our attention may be directed, in both, to the local cause of the disturbance of function;* the Doctor keeps this relative connexion in view throughout his work, and thus endeavours to advance the pathology of insanity in the same way as that of other diseases: in chronic affections of most other organs, he thinks we have greatly improved, *if not altogether derived*, our principles of treatment from observing the progress, and the remedies, of their acute diseases.

Having shewn that mere disturbance of a function is not an actual disease, but an effect of various and often opposite affections of the organ which performs it, and that mental derangement is not a specific disease, but a symptom of an actual cerebral affection, Dr. Combe concludes—that although the terms *mania, melancholia, insanity, idiocy*, and the like, may be used to designate the particular *mental* forms assumed by the symptoms, these terms ought to be entirely discarded as *names of diseases*, because their use serves to perpetuate the error, *that they really note specific states, requiring in all cases a specific treatment:* we ought rather to speak of the various *maladies* of the brain; just as in lesions of the lungs; *instead of regarding dyspnœa as a positive disease,* we go back to the local or organic affection, and speak of pneumonia, of pleuritis, or of phthisis, of which *impeded breathing* is nothing more than a symptom. In applying this principle to those affections of the brain which give rise to mental derangement, we shall at the first, from the excess of our ignorance, make a very poor appearance; but even in the attempt, says Dr. C. there will be the superlative advantages of keeping the very limited extent of our knowledge constantly before our eyes, and of stimulating us to unremitting exertion in the only path calculated to improve or increase it; whereas, he adds, it is no less true than melancholy, that the only use of our present nomenclature is to make us deceive ourselves, and rest satisfied with a *word* in the absence of an *idea*:—the method of naming the disease after the *prominent symptom,* without regard to the nature of the *organic cause,* lies at the root of all the confusion and contradiction that have encumbered the investigation of the cerebral affections which produce insanity.

By the evidences which prove *that the brain is an aggregate of organs, each manifesting a distinct mental power,* it is proved, also, that one or more of these organs may be injured or diseased, and their functions impeded or altered, without necessarily affecting the remainder. This explains *how* a person may be insane on one feeling or faculty, and sound on all the rest; and, consequently, *how*, when a different organ is diseased, the faculty or feeling that is deranged may be different, and the prominent symptoms be different, and yet the *disease* itself remain exactly of the same nature. Inflammation affecting the eye disturbs vision, because vision is the eye's *function;* and, affecting the ear, it disturbs hearing, because hearing is the ear's *function;* still, however, it is *inflammation* in both organs, and, in both, it requires the same *kind* of treatment. In like manner, morbid excitement of certain parts of the brain may produce raving, violence, and fury; and morbid excitement of other parts may occasion fear, despondency, and melancholy; now this does not proceed from any difference in the *kind* of excitement; it proceeds from the circumstance, that each of these different parts executes a peculiar and different *function;* hence, also, it is, that both cases may require a similar *kind* of medical treatment; and, in so far, this view of the subject affords a simple and consistent explanation of all the various forms which insanity assumes, and leaves us free to observe with care, the *nature* of the organic lesion on which each *kind* of symptoms depends. Viewing the question conversely, on the principle—*that the brain is a* UNIT, *the* whole *of which serves* equally *to manifest* all *the mind's faculties,* we shall perceive the utter impossibility of explaining how it happens that, in a majority of instances, a *few* only of the mental powers are deranged, while the rest continue unaffected. If the *whole* brain were the *single* organ of mind, every part of it ought to concur in every mental operation, and all the faculties of mind—*whereof the brain is*

*held to be the* sole *instrument*—ought, in *every case*, to be equally deranged, and the patient ought to pass rapidly and often from an abyss of despondency to the abodes of bliss, or from a state of listless apathy to that of demoniacal fury. This, to be sure, is sometimes actually the case; but it is far more rare than that in which the mental affection is *partial*, and retains its characteristical features *unchanged*. The idiot, who to-day manifests strongly the sentiment of benevolence, or of veneration, or of pride, will not to-morrow, nor in a year, change the nature of his predominant manifestations; neither will the monomaniac, who to-day fancies himself a king, or possessed of boundless power and wealth, believe himself to-morrow a slave, or in wretchedness and want: nor will the rich lunatic, whose fear is that of dying from starvation, manifest the gaiety of one who considers himself the favourite of some supernatural power: such transitions of fancy, however, might have been expected—*had the brain existed as a* unit, *the* sole *organ of* all *the mental faculties*. Heterogeneous manifestations, and rapid changes from one class of ideas to another, do, indeed, sometimes occur, but then the *whole* brain, including *all* the organs, is diseased; this state, therefore, affords a true picture of the nature of insanity—such as it would necessarily be, in every instance, if the organ of the mind were SINGLE. So long, then, as we continue to advocate the *unity* of the organ of mind, we shall be constrained—*in accounting for the variety of forms which derangement of so many mental faculties and organs may assume*—to create a new malady for every change in the appearance of the mental symptoms; and, following the wide variety thus presented, to conjure up a list of mental diseases, numerous and complicated enough to damp the ardour of the most diligent and determined student, and, at the same time, running so much into each other, as to defy all attempts at discriminating or describing them. We may avoid falling into this portentous error, however, by investigating the *chronic* affections of the brain, in the same way as we study its more rapid and *acute* diseases. Regarding the delirium or mental derangement attending the *latter*, as a mere *symptom* only—*and a most important one it is in the indications*—we make use of it, and of all other means, to detect the *nature* of the organic affection; and, *by this last*, are guided in the application of our remedies. Let us follow the same course with the mental aberration, which forms so striking a feature of the *chronic* affection, and, ultimately, our success will convince us that we have entered upon the right road to improvement.

In another article, we shall complete our analysis of this very elaborate and ingenious work, which is not so much known in the profession as it deserves to be.

---

